# Multilayered insights: a machine learning approach for personalized prognostic assessment in hepatocellular carcinoma

**DOI:** 10.3389/fonc.2023.1327147

**Published:** 2024-02-29

**Authors:** Zhao-Han Zhang, Yunxiang Du, Shuzhen Wei, Weidong Pei

**Affiliations:** ^1^ Shenyang No.20 High School, Shenyang, China; ^2^ Department of Oncology, Huai’an 82 Hospital, China RongTong Medical Healthcare Group Co., Ltd., Chengdu, China; ^3^ Department of Discipline Development, China RongTong Medical Healthcare Group Co., Ltd., Chengdu, China

**Keywords:** hepatocellular carcinoma, prognosis risk model, machine learning, immune function, drug responsiveness

## Abstract

**Background:**

Hepatocellular carcinoma (HCC) is a complex malignancy, and precise prognosis assessment is vital for personalized treatment decisions.

**Objective:**

This study aimed to develop a multi-level prognostic risk model for HCC, offering individualized prognosis assessment and treatment guidance.

**Methods:**

By utilizing data from The Cancer Genome Atlas (TCGA) and the Surveillance, Epidemiology, and End Results (SEER) database, we performed differential gene expression analysis to identify genes associated with survival in HCC patients. The HCC Differential Gene Prognostic Model (HCC-DGPM) was developed through multivariate Cox regression. Clinical indicators were incorporated into the HCC-DGPM using Cox regression, leading to the creation of the HCC Multilevel Prognostic Model (HCC-MLPM). Immune function was evaluated using single-sample Gene Set Enrichment Analysis (ssGSEA), and immune cell infiltration was assessed. Patient responsiveness to immunotherapy was evaluated using the Immunophenoscore (IPS). Clinical drug responsiveness was investigated using drug-related information from the TCGA database. Cox regression, Kaplan-Meier analysis, and trend association tests were conducted.

**Results:**

Seven differentially expressed genes from the TCGA database were used to construct the HCC-DGPM. Additionally, four clinical indicators associated with survival were identified from the SEER database for model adjustment. The adjusted HCC-MLPM showed significantly improved discriminative capacity (*AUC*=0.819 *vs.* 0.724). External validation involving 153 HCC patients from the International Cancer Genome Consortium (ICGC) database verified the performance of the HCC-MLPM (*AUC*=0.776). Significantly, the HCC-MLPM exhibited predictive capacity for patient response to immunotherapy and clinical drug efficacy (*P* < 0.05).

**Conclusion:**

This study offers comprehensive insights into HCC prognosis and develops predictive models to enhance patient outcomes. The evaluation of immune function, immune cell infiltration, and clinical drug responsiveness enhances our comprehension and management of HCC.

## 1 Introduction

Primary liver cancer, a prevalent malignancy of the digestive system, ranks as the sixth most frequently occurring tumor globally and is the second leading cause of mortality (1, 2). Hepatocellular carcinoma (HCC) is the prevailing pathological subtype of primary liver cancer, accounting for 75%-85% of all cases (3). The poor prognosis of HCC arises from its early propensity for metastasis, often involving dissemination to the portal vein or distant organs (4). Patients with early-stage HCC have access to a potentially curative treatment option with a long-term survival rate exceeding 5% at 60 years, while patients with advanced-stage tumors experience a median survival period ranging from 1 to 2 years (5–7). Therefore, timely identification, early intervention, and the implementation of rational and effective treatment strategies are crucial for patients diagnosed with HCC (8).

Surgical resection is considered represents the primary therapeutic approach for patients with early-stage HCC and often leads to favorable outcomes (9, 10). However, for individuals diagnosed with intermediate or advanced-stage HCC, surgical resection is no longer a feasible option due to tumor progression and metastasis. Local regional therapies, such as ablation, arterial-directed therapies, or external beam radiation therapy, are the preferred treatment modalities for patients with localized liver disease that cannot be surgically removed or are not suitable for surgery. Systemic therapies are recommended for patients who undergo disease progression after local regional therapies or those with metastases outside the liver (11). This focus on systemic therapies highlights the importance of considering the tumor microenvironment, drug responsiveness, and immunotherapy as crucial factors (12–14). Targeted therapies are particularly relevant for patients diagnosed with intermediate or advanced-stage HCC (15, 16). Sorafenib, initially approved for advanced HCC treatment, is hindered by the development of resistance (17, 18). Subsequently, other multi-kinase inhibitors, such as Lenvatinib, Regorafenib, Cabozantinib, and the VEGFR2 inhibitor ramucirumab, have been approved as second-line targeted treatment options (19). With a deeper understanding of the interplay between the immune system and cancer, immune checkpoint inhibitors (ICI) have been integrated into the therapeutic arsenal for patients with advanced HCC. Nivolumab, an ICI agent, has been FDA approval for the management of advanced HCC (20, 21). Given the expanding range of treatment methods, the selection of the most suitable treatment plan for patients has become critical.

Therefore, to provide optimal treatment approaches for different stages of HCC progression, conventional methods often assign patients to specific stages based on the Barcelona Clinic Liver Cancer (BCLC) classification (6, 22). BCLC staging, widely utilized in HCC, categorizes patients into different stages based on factors such as tumor size, number, liver function, and symptoms (23). Treatment strategies, such as surgical resection, liver transplantation, radiofrequency ablation, radiation therapy, and targeted therapy, are determined for patients according to their corresponding stages (6). However, significant heterogeneity exists among patients, including genetic variations, immune environments, and tumor heterogeneity. Relying solely on conventional staging methods may insufficiently consider individual patient characteristics and the complexities of the disease, potentially leading to inaccurate prognosis assessments and suboptimal personalized treatments.

Recent advancements in tumor genomics research have facilitated the utilization of extensive tumor genomic data to gain insights into the complexity and individual variations of tumors. The Cancer Genome Atlas (TCGA) database, as a comprehensive repository of diverse cancer-related data, offers new opportunities for exploring prognostic risk assessment in HCC (24, 25). Therefore, in contrast to conventional approaches, we consider incorporating factors such as molecular biology information and the tumor microenvironment based on clinical indicators. By leveraging the potential of powerful machine learning and big data analysis techniques, we can extract valuable insights from extensive tumor genomic data to construct prognostic risk assessment models.

Through a comprehensive analysis of clinical indicators, molecular biology information, tumor microenvironment, and other multi-level factors, o our objective is to establish a comprehensive and accurate HCC prognostic risk model and explore its association with drug responsiveness and immunotherapy (26, 27). By improving the accurate assessment of prognostic risk in HCC patients, we can provide essential evidence to inform the development of personalized treatment plans, thus improving prognosis assessment and treatment outcomes for patients. Moreover, by leveraging the extensive resources of databases such as TCGA and SEER, this study has the potential to make significant breakthroughs and advancements in the field of HCC prognostic assessment and personalized treatment.

## 2 Research design and methods

### 2.1 Research workflow

**Figure 1:**
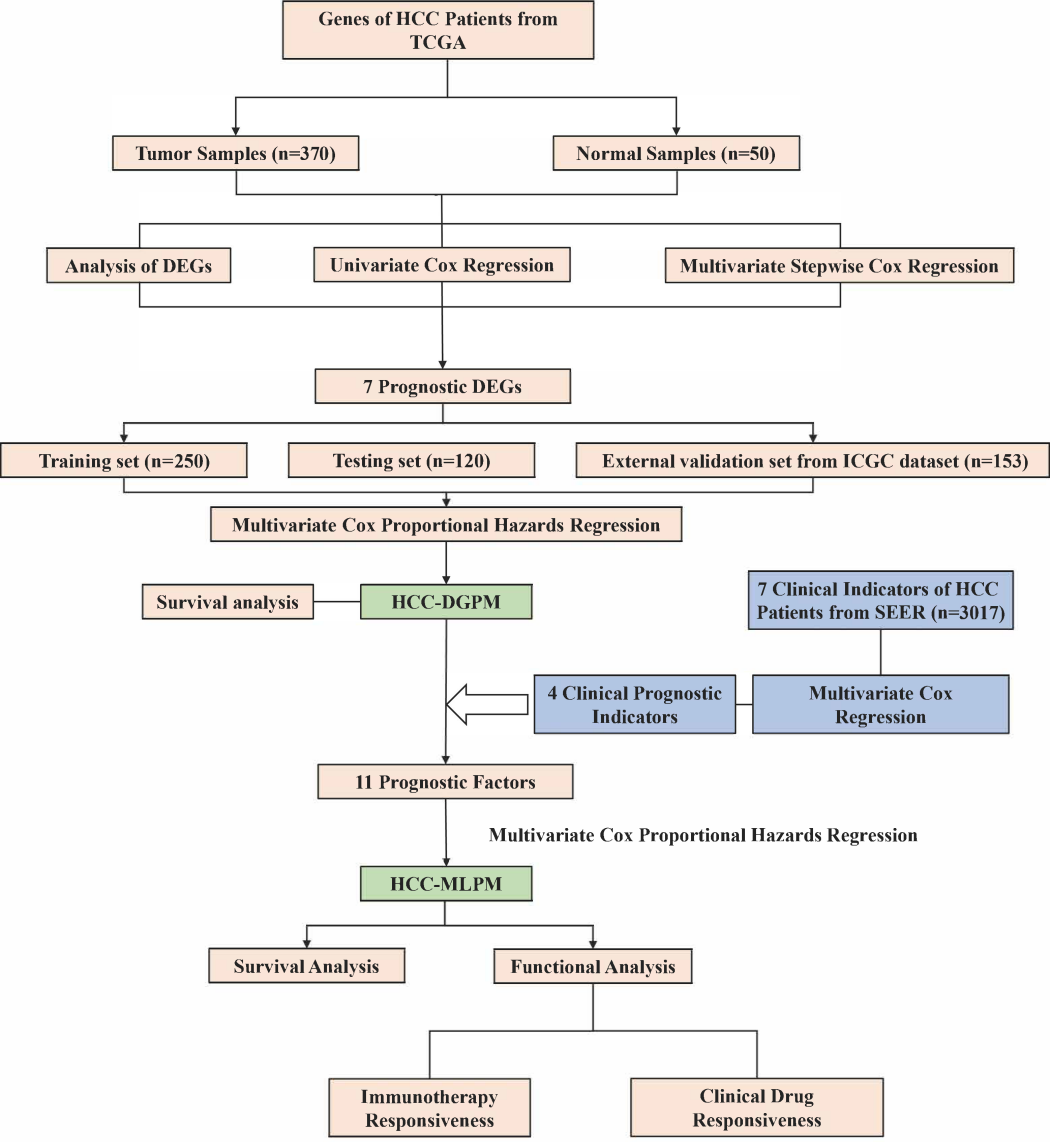
Research workflow for the construction of hepatocellular carcinoma prognostic model. HCC, Hepatocellular carcinoma; TCGA, The Cancer Genome Atlas; SEER, Surveillance, Epidemiology, and End Results; DEGs, Differentially expressed genes; HCC-DGPM, HCC Differential Gene Prognostic Model; HCC-MLPM, Hepatocellular Carcinoma Multilevel Prognostic Model.

The research workflow (depicted in **Figure 1**) comprised the subsequent steps: 1) Identification of differentially expressed genes (DEGs) associated with survival through gene expression analysis; 2) Development of the HCC Differential Gene Prognostic Model (HCC-DGPM) by integrating significant DEGs; 3) Selection of clinical indices linked to survival; 4) Model adjustment and validation, culminating in the HCC Multilevel Prognostic Model (HCC-MLPM); 5) Evaluation of immune function and analysis of clinical drug responsiveness.

### 2.2 Data collection and preparation

The dataset for liver cancer was obtained from the TCGA database through the website, and the gene expression matrices of adjacent non-cancerous and cancerous tissues were used for the analysis. The dataset consisted of 50 samples of normal liver tissue and 370 samples of liver cancer. Additionally, data from 30,684 patients diagnosed with primary liver cancer between 1988 and 2015 were extracted from the SEER database using SEER*stat software 8.4.0 (https://seer.cancer.gov/). After data cleansing, a total of 3,017 patient records were available for further analysis. The International Cancer Genome Consortium (ICGC) database provided data from 369 HCC patients for the study. After further data cleaning, cases with incomplete clinical information were excluded, resulting in a final sample size of 153 cases.

It is important to note that all the included patients were diagnosed with primary liver cancer. The year of the initial diagnosis was categorized into 5-year intervals and considered as an ordinal variable. Age 45 was chosen as the threshold to classify cases with early-onset HCC, and age was divided into 10-year intervals.

### 2.3 Selection of HCC prognostic DEGs

The “limma” package in R was used to identify the DEGs between cancerous and adjacent non-cancerous tissues (28). The DEG threshold was set as an absolute log2-fold change (FC) ≥ 1 and an adjusted *P* < 0.05. Volcano plots illustrating the DEGs were generated using the “ggplot2” package (https://ggplot2.tidyverse.org/). Subsequent screening involved conducting both univariate and stepwise multivariate Cox regression analyses. In the univariate Cox regression analysis, each DEG was evaluated individually to assess its association with the survival outcome. In this context, the survival outcomes were solely considered as “death”. This analysis facilitated the identification of genes that exhibited a significant correlation with patient survival. Subsequently, a stepwise multivariate Cox regression analysis was performed.

### 2.4 Construction and validation of HCC-DGPM

The patients from the TCGA dataset were randomly assigned to training (n = 250) and testing (n = 120) sets in a 7:3 ratio, facilitated by the “caret” R package for random assignment (29). The training set was used to train the model, while the testing set was used to assess the predictive performance of the model. HCC-DGPM was constructed using the multivariate Cox regression method. External validation set was performed using the ICGC dataset (n = 153). The performance of HCC-DGPM was evaluated using receiver operating characteristic (ROC) curves, with a higher area under the curve (AUC) indicating improved predictive accuracy. To enhance the precision of our prognostic model, calibration curves were utilized, employing the “rms”, “survival”, and “ResourceSelection” R packages (https://CRAN.R-project.org/package). These curves are a measure of how closely the model’s predictions align with actual outcomes. The closer these curves lie to the 45-degree line, the more accurate the model is, indicating a high degree of concordance between predicted and observed results. The HCC-DGPM was validated with Kaplan-Meier (KM) curves, and the methodology for establishing the cutoff value for risk groups was not initially specified. The cutoff value used for delineating high and low risk groups was determined by the method that maximizes (sensitivity + specificity - 1). The established cutoff value for the risk score was 1.65.

### 2.5 Model adjustment and validation

To improve the model’s performance, we performed multivariate Cox regression analysis to identify clinical indicators associated with HCC patient survival using the SEER database. SSubsequently, these indicators were used to refine the HCC-DGPM, resulting in the HCC-MLPM. ROC curves and KM curves were generated to assess the performance of the HCC-MLPM and provide additional validation of its effectiveness.

### 2.6 Immune evaluation of the model

Gene Set Enrichment Analysis (GSEA) was performed to investigate the impact of the risk score on the biological function of HCC patients. The annotated gene setlist was selected using a significance threshold of *P* < 0.05. Moreover, the “ssGSEA” R package was used to estimate the infiltration levels of 28 distinct immune cell types in HCC patients (30), taking into account their risk scores. The IPS was used to assess patient responsiveness to immunotherapy (31).

### 2.7 Clinical drug responsiveness evaluation

To explore the variations in clinical drug responsiveness among patients, we analyzed the clinical drug information and patients’ drug responsiveness data retrieved from the TCGA database. We evaluated the effects of chemotherapy drugs such as Gemcitabine, Cisplatin, Doxorubicin, 5-fluorouracil (5-FU), Oxaliplatin, Adriamycin, and Cytoxan, alongside targeted therapy agents including Sorafenib, Everolimus, Sunitinib, and Temsirolimus. This comprehensive assessment was crucial as it is well-recognized that therapeutic efficacy varies significantly between treatments, independent of other evaluated variables.

Based on the risk scores generated by our model, patients were categorized into high-risk and low-risk groups. Subsequently, a proportional stacked bar chart was used to visually represent and analyze the disease progression in these two patient groups.

### 2.8 Statistical analysis

Cox regression models were employed to calculate hazard ratios (HR) and assess the relationship between gene expression and survival outcomes. The KM method was utilized to generate survival curves, and the log-rank test was applied to compare these curves. The Jonckheere-Terpstra test and Cochran-Mantel-Haenszel test were performed to evaluate the trend association between the diagnosis year and patient characteristics for numerical and categorical data, respectively. Cox proportional hazards regression models were used to HRs and their corresponding 95% confidence intervals for prognostic factors related to overall survival (OS). In the multivariate Cox regression analyses, a stepwise procedure was conducted with an entry criterion of *P* < 0.05 to identify the most statistically significant prognostic factors. The significance level for all statistical tests was set at *P* < 0.05. Statistical analyses were conducted using SAS 13.2 (SAS Institute, Cary, NC, USA), and the KM curves were plotted using R Software.

### 2.9 Availability of data

The data utilized in this study can be obtained by contacting the authors due to restrictions imposed by the data providers, namely TCGA, SEER, and GSEA databases. Access to these databases is available via their dedicated websites: TCGA (https://portal.gdc.cancer.gov/), SEER (https://seer.cancer.gov/), GSEA (http://software.broadinstitute.org/gsea/index.jsp), and ICGC (https://icgcportal.genomics.cn/). Researchers interested in accessing the data may reach out to the authors for additional information and support in acquiring the required permissions and data access.

## 3 Results

### 3.1 Analysis of differential gene expression

**Figure 2:**
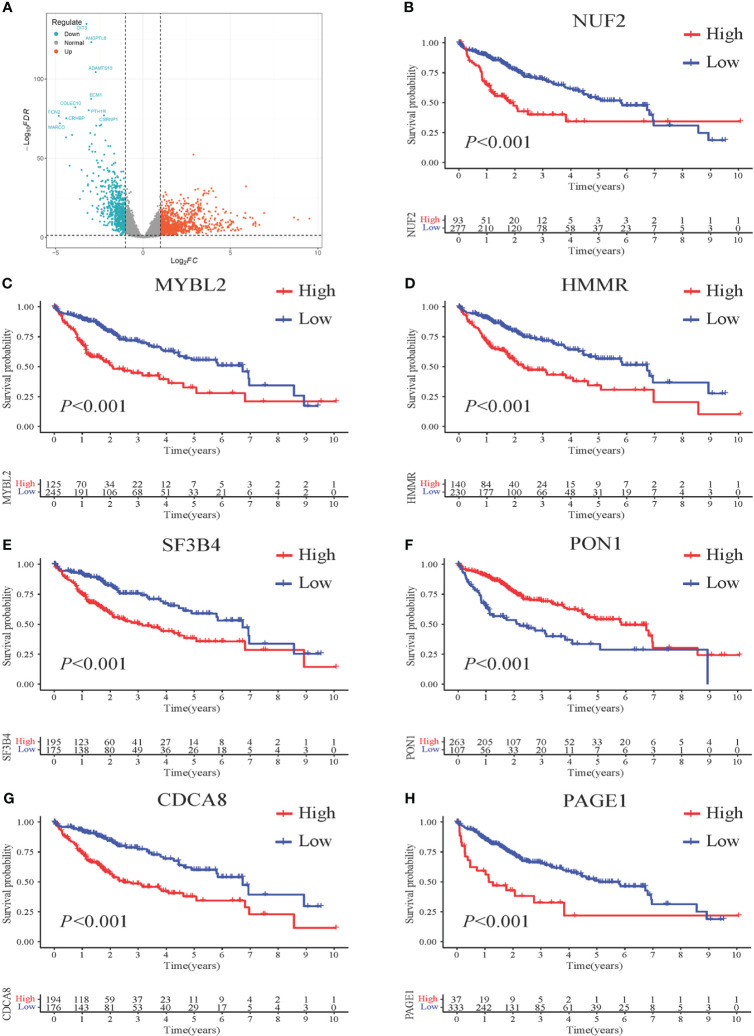
Survival impact of 7 DEGs screened from HCC patients. **(A)** Volcano plots illustrating the DEGs from HCC *vs*. normal. Genes upregulated in HCC are represented in red, while genes downregulated in HCC are shown in blue. The x-axis represents the log2-fold change in gene expression, indicating the magnitude of change, and the y-axis represents the statistical significance (-log10 p-value) of the differential expression. **(B–H)** Kaplan-Meier (KM) curves demonstrating the association between gene expression levels and patient survival. High-risk patients are depicted in red, while low-risk patients are represented in blue. The x-axis represents the survival time, and the y-axis represents the survival probability.

To establish a prognostic model, 370 HCC samples and 50 samples of normal liver tissue from TCGA were involved. Compared to normal group, a total of 1761 DEGs were identified, comprising 1,091 upregulated genes and 670 downregulated genes in HCC group. The expression patterns of these DEGs are illustrated in **Figure 2A** through volcano plots.

### 3.2 Identification of genes associated with patient prognosis

To further identify the prognostic genes, a univariate Cox regression was performed on the 1,761 genes to identify genes significantly associated with patient prognosis. This analysis yielded a subset of 89 genes that showed a significant association at a significance level of *P* < 0.001. Further analysis using multivariate Cox stepwise regression identified seven genes significantly associated with the survival of HCC patients: MYBL2, SF3B4, CDCA8, NUF2, HMMR, PON1, and PAGE1 (**Table 1**).

**Table 1:** Differential genes associated with OS in HCC patients.

Name	HR	HR.95L	HR.95H	*P value*
MYBL2	0.4820	0.31979	0.7264	0.000488
SF3B4	3.0446	1.90062	4.8770	3.63e-06
CDCA8	3.1851	1.65058	6.1463	0.000552
NUF2	0.1932	0.08874	0.4205	3.43e-05
HMMR	3.1205	1.73930	5.5985	0.000136
PON1	0.7698	0.67562	0.8771	8.47e-05
PAGE1	1.2691	1.11467	1.4448	0.000318

KM analysis was performed to assess the impact of the seven identified genes on patient survival. The results revealed a significant correlation between these genes and patient prognosis, as evidenced by distinct survival patterns observed in patient groups with high and low expression levels of these genes. Moreover, the survival analysis demonstrated that patients with higher gene expression levels had a significantly poorer prognosis compared to those with lower expression levels (**Figures 2B–H**).

### 3.3 Construction and validation of the HCC-DGPM

Therefore, HCC-DGPM was constructed through univariate Cox proportional hazards regression analysis using the expression levels of the seven identified genes (MYBL2, SF3B4, CDCA8, NUF2, HMMR, PON1, and PAGE1) as covariates. The regression coefficients were used to assign weights to each gene, allowing for the development of a risk score formula to calculate the individual risk score for each patient The risk score formula is defined as follows: Risk score = (Expression level of MYBL2 × 0.4820) + (Expression level of SF3B4 × 3.0446) + (Expression level of CDCA8 × 3.1851) + (Expression level of NUF2 × 0.1932) + (Expression level of HMMR × 3.1205) + (Expression level of P0N1 × 0.7698) + (Expression level of PAGE1 × 1.2691).

**Figure 3:**
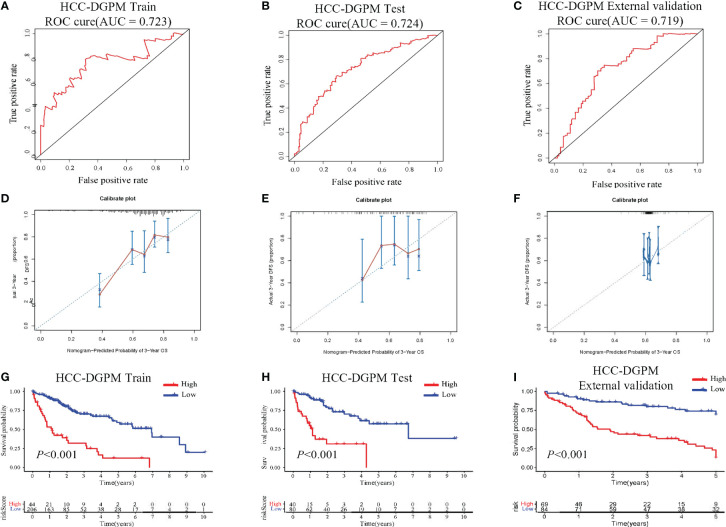
Performance Evaluation of the HCC-DGPM. **(A)** ROC curve of the training dataset. **(B)** ROC curve of the testing set. **(C)** ROC curve of the external validation set. The x-axis represents the false-positive rate, while the y-axis represents the true-positive rate. **(D–F)** Calibration curves for the training, testing, and external validation sets, respectively. **(G–I)** KM curves for overall survival (OS) in the training, testing and external validation set sets respectively (red: High risk; blue: Low risk).

ROC curve was performed to assess the predictive ability of the HCC-DGPM in determining patient outcomes. The training dataset exhibited an AUC of 0.723 (**Figure 3A**) for the HCC-DGPM, while the testing and external validation sets showed AUC values of 0.724 & 0.719 (**Figures 3B, C**). These results suggest that the HCC-DGPM has a moderate predictive ability to distinguish between high-risk and low-risk patients. To enhance the credibility of our model’s accuracy, we performed calibration curve analyses following the ROC assessments (**Figures 3D–F**). The results from these calibration curves lend further credence to the model’s predictive acumen, highlighting its prospective value in a clinical setting.

In addition, survival analysis was conducted based on the newly calculated risk score, allowing for the classification of patients into high-risk and low-risk groups for model validation purposes. The KM curves demonstrated significant differences in survival between the high-risk and low-risk groups (**Figures 3G–H**).

### 3.4 Model adjustment

Clinical indicators, including Age, Race, Sex, tumor size (T), node involvement (N), metastasis (M), and stage, were screened from the SEER database due to their potential correlation with patient survival in HCC. Four indicators were identified as significantly associated with survival outcomes (**Table 2**). Multivariate Cox regression analysis was conducted to determine the clinical factors significantly associated with patient survival. Four indicators were found to be significantly correlated with survival outcomes (**Table 2**). Next, the identified clinical indicators from the SEER database were integrated with the risk scores obtained from the 7 DEGs, resulting in the development of a novel predictive model (HCC-MLPM). The adjusted predictive HCC-MLPM is represented by the following formula: Risk Score = (Expression level of MYBL2 × 0.4820) + (Expression level of SF3B4 × 3.0446) + (Expression level of CDCA8 × 3.1851) + (Expression level of NUF2 × 0.1932) + (Expression level of HMMR × 3.1205) + (Expression level of PON1 × 0.7698) + (Expression level of PAGE1 × 1.2691) + (Age × 1.5079) + (T × 2.9376) + (N × 0.8721) + (M × 3.0453).

**Table 2:** Risk factors in the SEER database.

risk factors	HR	HR.95L	HR.95H	*P value*
Age	1.5079	1.2375	1.8374	4.63e-05
T	2.9376	2.5259	3.4165	2e-16
N	0.8721	0.7641	0.9954	0.0425
M	3.0453	2.4834	3.7343	2e-16

### 3.5 Evaluation of HCC-MLPM

**Figure 4:**
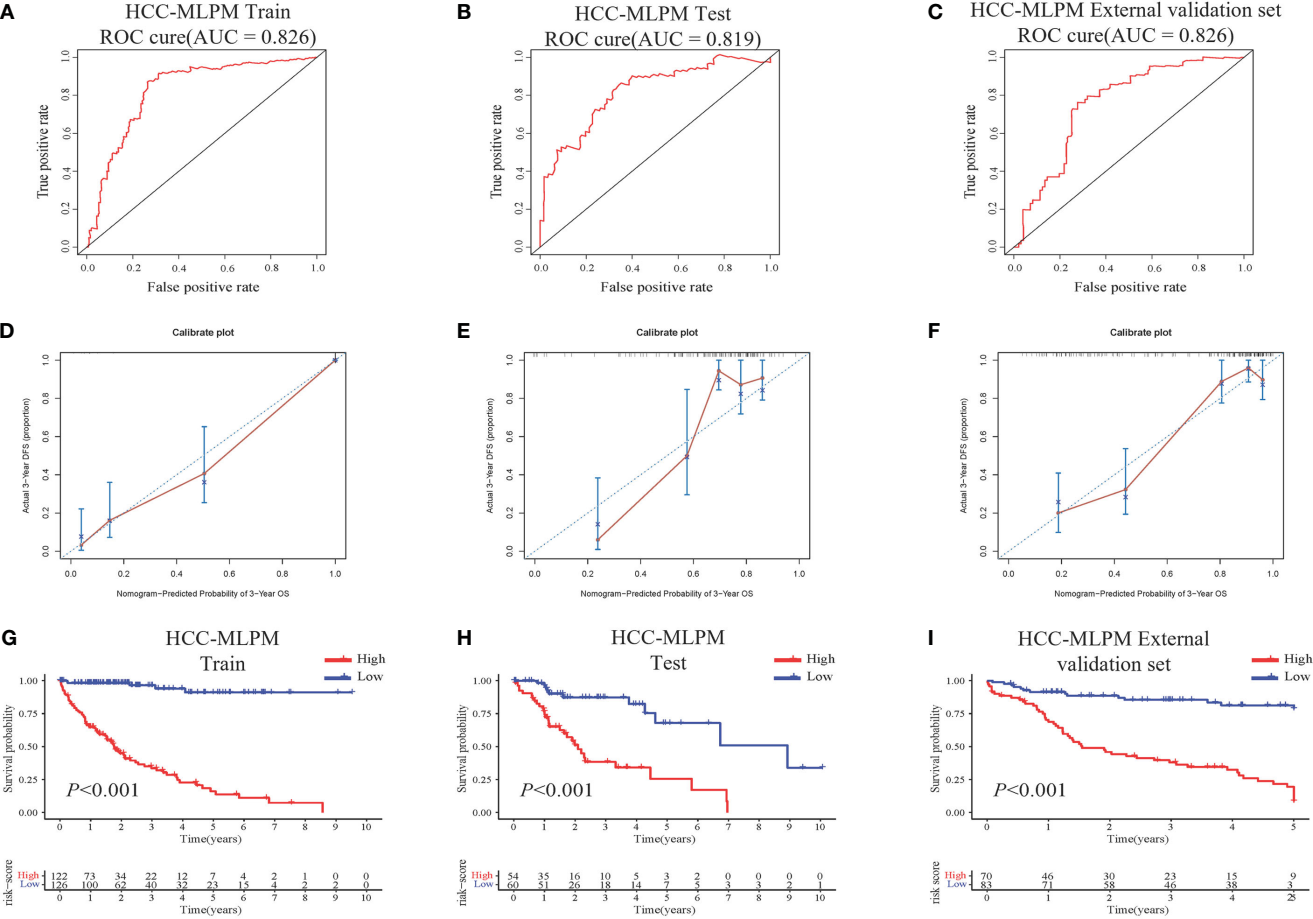
Performance Evaluation of the HCC-MLPM. **(A)** ROC curve of the training dataset. **(B)** ROC curve of the testing set. **(C)** ROC curve of the external validation set. **(D–F)** Calibration curves for the training, testing, and external validation sets, respectively. **(G–I)** KM survival curves for OS of the training, testing and external dataset respectively (red: High risk; bule: Low risk).

The performance of the HCC-MLPM was evaluated using both the training and testing datasets. In the training dataset, the HCC-MLPM demonstrated improved predictive ability with an AUC of 0.826 (**Figure 4A**). In the testing dataset, the HCC-MLPM achieved an AUC of 0.819 (**Figure 4B**), further validating its enhanced predictive capacity. Similarly, the HCC-MLPM exhibited satisfactory predictive capability in the external validation dataset, achieving an AUC of 0.776 (**Figure 4C**). These results indicate that the HCC-MLPM effectively discriminates between high-risk and low-risk patients in this external validation set. Calibration curves were subsequently integrated, serving as an additional verification stratum for the model’s validity (**Figures 4D–F**).

Furthermore, by utilizing the scoring system derived from the adjusted model, patients were categorized into high-risk and low-risk groups. KM curves showed that patients in the low-risk group had superior overall survival outcomes compared to those in the high-risk group (**Figures 4G–H**). These findings indicate that the integration of risk scores based on differential gene expression, along with the selected clinical indicators, significantly improved the predictive performance of the HCC-MLPM.

### 3.6 Stratified survival analysis based on clinical indicators

**Figure 5:**
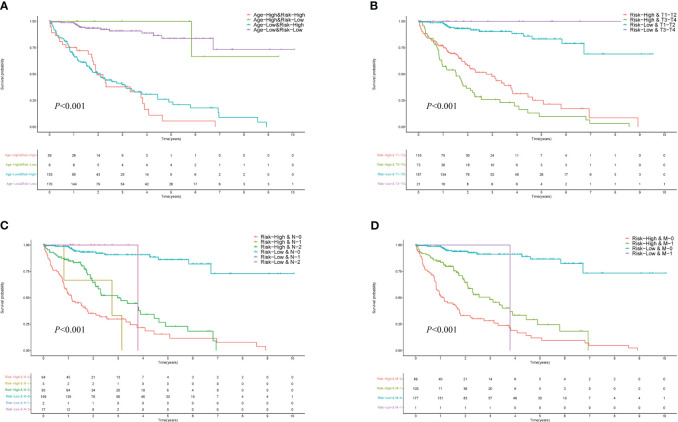
Performance for Different Clinical Indicators in HCC-MLPM. **(A)** KM curves for Age. **(B)** KM curves for tumor size (T). **(C)** KM curves for node involvement (N). **(D)** KM curves for metastasis (M).

This section delves into a detailed survival analysis of HCC patients within the HCC-MLPM framework, stratified according to key clinical indicators. The KM curves display distinct survival probabilities over time for groups stratified by key clinical indicators: Age (**Figure 5A**), T (**Figure 5B**), N (**Figure 5C**), and M (**Figure 5D**). These curves reveal considerable variation in survival outcomes across these different clinical stratifications (*P*<0.001), underscoring the significant impact of each indicator on survival. They highlight the potential utility of these clinical indicators in refining the HCC-MLPM.

### 3.7 Gene set enrichment analysis

**Figure 6:**
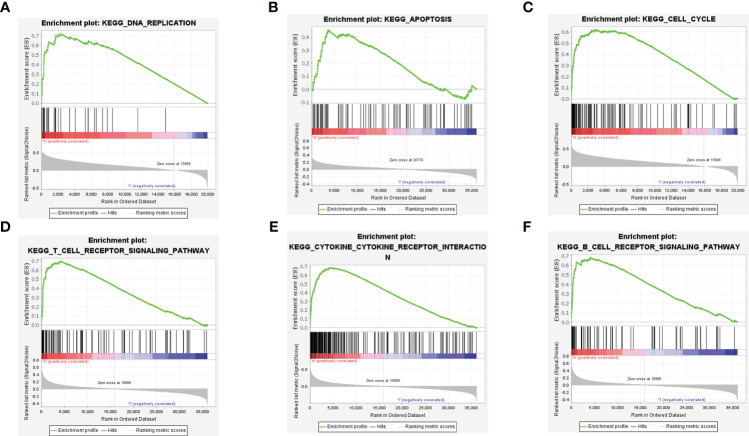
Gene Set Enrichment Analysis (GSEA) of the HCC-MLPM. **(A–C)** DNA replication, apoptosis and cell cycle enrichment analysis by GSEA. **(D-F)** T and B cell receptor pathway, cytokine receptor interaction pathway enrichment analysis by GSEA.

To assess the immune function associated with the HCC-MLPM, Gene Set Enrichment Analysis (GSEA) was performed. The analysis revealed that high-risk patients showed a stronger association with cellular processes related to the cell cycle and DNA replication, indicating a more aggressive tumor phenotype compared to low-risk patients (**Figures 6A–C**). Moreover, the high-risk group exhibited a closer association with immune response compared to the low-risk group. This was evident from the enrichment of gene sets related to the toll-like receptor signaling pathway, cytokine-cytokine receptor interaction, and chemokine signaling pathway. These findings highlight a significant correlation between the risk score and the immune status of HCC (**Figures 6D–F**).

### 3.8 Immune assessment of the HCC-MLPM

**Figure 7:**
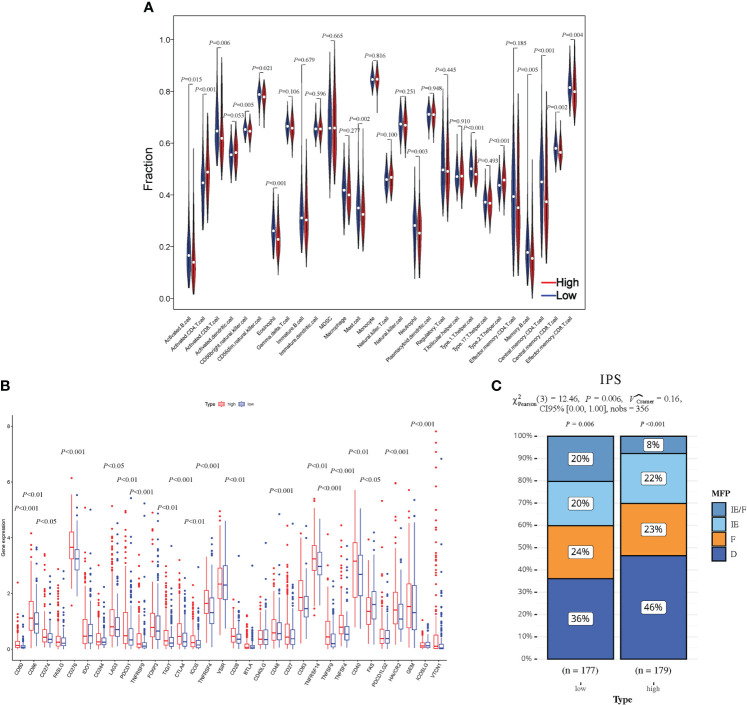
Evaluation of the HCC-MLPM on tumor immunity. **(A)** The Violin plots for infiltration levels of specific immune cell types. **(B)** Boxplot for infiltration levels of immune checkpoint markers. **(C)** The performance for immunotherapy. IE/F, Immune-enriched/Fibrotic; IE, Immune-enriched; F, Fibrotic; D, Depleted; High, high risk; Low, low risk. (red: High risk; bule: Low risk).

Using the single-sample Gene Set Enrichment Analysis (ssGSEA) method, we conducted an analysis of immune cell infiltration in HCC patients, comparing the high-risk and low-risk groups. Violin plots further illustrated significantly lower infiltration levels of activated B cells, activated CD8+ T cells, natural killer cells, immature B cells, mast cells, and memory CD4+ T cells in the high-risk group. Conversely, the infiltration level of activated CD4+ T cells was significantly higher in the high-risk group (**Figure 7A**). Additionally, we examined the expression changes of immune checkpoint markers between the high-risk and low-risk groups. Remarkably, the high-risk group exhibited a significant upregulation in the expression levels of most immune checkpoint markers (**Figure 7B**). These findings indicate that the high-risk group of HCC patients displays lower levels of immune cell infiltration, particularly in specific immune cell subsets, along with higher expression of immune checkpoint markers. These observations suggest the presence of a potentially immunosuppressive microenvironment in the high-risk group, which may contribute to disease progression and poorer prognosis.

To assess the responsiveness of patients in both groups to immunotherapy, we utilized an unsupervised clustering approach based on the characteristics of the tumor microenvironment, specifically the IPS. The patients were categorized into four groups: immune-enriched/fibrotic (IE/F), immune-enriched (IE), fibrotic (F), and immune-depleted (D). Among these groups, IE/F and IE demonstrated a more favorable response to immunotherapy, while F and D were associated with relatively poorer responses. In our analysis, we observed a higher proportion of patients in the low-risk group with an IE/F microenvironment. However, the proportions of IE and F were comparable between the two patient groups (IPS) (**Figure 7C**).

### 3.9 Assessment of clinical drug responsiveness

**Figure 8:**
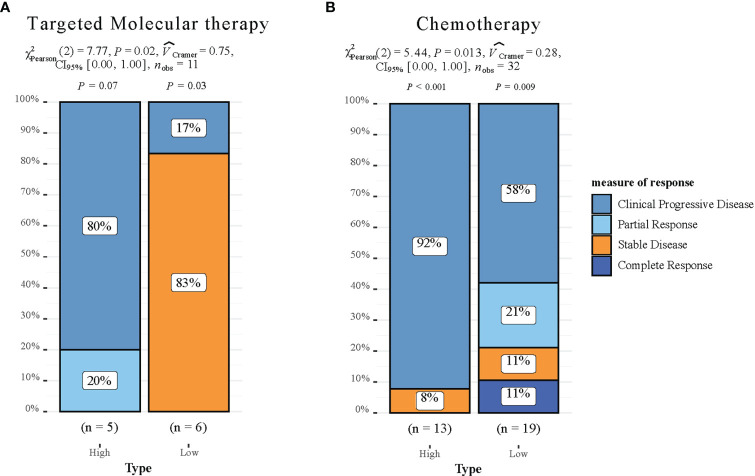
Drug responsiveness Evaluation of the HCC-MLPM. **(A)** Performance for predicting targeted molecular therapy of HCC-MLPM. **(B)** Performance for chemotherapy of HCC-MLPM. (High: high risk; Low: low risk).

To investigate the clinical drug responsiveness predicted by HCC-MLPM, we utilized drug information obtained from the TCGA database. We examined the correlation between drug targets (Sorafenib) and cytotoxic drugs with the risk scores derived from our model. The results revealed significant differences in drug responsiveness between the high-risk and low-risk groups. Notably, a higher proportion of patients in the low-risk group demonstrated disease stability when treated with both targeted therapy and chemotherapy regimens (**Figures 8A, B**). This finding suggests that these treatment approaches were more likely to be effective in the low-risk group, providing potential therapeutic options for this particular subgroup of patients.

## 4 Discussion

We have successfully developed a machine learning-based prognostic risk model specifically designed for patients with HCC. This study integrates diverse indicators from a multicenter dataset, providing a comprehensive tool to aid in personalized treatment decisions for HCC patients. Remarkably, this model accurately predicts patient survival outcomes and offers insights into the effectiveness of immunotherapy and other clinical drug treatments in HCC patients.

The molecular pathogenesis of HCC is highly complex and heterogeneous. Currently, clinical treatment decisions for HCC patients are primarily based on limited clinical and pathological indicators (32). However, the development and progression of HCC are influenced by various factors, such as genetic variations, aberrant cell signaling pathways, and alterations in the immune environment (33, 34). Therefore, there is an urgent need to develop a dependable prognostic risk assessment model to enable personalized treatment for HCC. Previous studies have predominantly focused on individual tumor indicators, including age, stage, pathological type, tumor size, and other clinical factors, which have been extensively utilized in clinical assessments (35, 36). With advancements in genomics and immunology, there is a growing emphasis on the molecular characteristics of HCC and the impact of the immune environment on patient prognosis. Researchers have made efforts to predict patient prognosis from a biological perspective by integrating diverse information sources, such as gene expression data, protein expression data, and immune cell infiltration (37, 38). However, these evaluation methods provide a limited perspective on patient prognosis, resulting in inherent limitations. In contrast, our study not only takes into account clinical and pathological indicators that reflect the overall patient condition and disease severity but also places significant emphasis on the biological characteristics of liver cell tumors. This approach involves the identification of differentially expressed genes in HCC and the unveiling of potential biological mechanisms. By utilizing a modeling approach that incorporates comprehensive multi-level indicators, our model can offer a more comprehensive and dependable prognostic risk assessment for HCC patients (39, 40). Similar methodologies have demonstrated favorable outcomes in studies focusing on diverse cancer types, underscoring their potential utility in personalized medicine. Specifically, researchers investigating breast cancer, bladder cancer, and colorectal cancer have achieved robust predictive results by integrating comprehensive models with diverse datasets encompassing clinical, gene expression, and proteomic information (41–43). These investigations additionally validate the feasibility of our modeling approach.

In terms of methodology, our research has made significant advancements. Cox regression, an advanced machine learning technique, has provided strong technical support. Machine learning, in comparison to traditional statistical methods, excels in managing complex data structures and relationships, facilitating the extraction of potential features and patterns from extensive clinical and gene expression data (41). By employing the feature selection and optimization process of Cox regression, we have identified the most relevant indicators for prognosticating HCC patients. This approach effectively reduces the dimensionality of the feature space and enhances the predictive performance of the model (AUC = 0.724 *vs*. 0.819). Importantly, our research leverages the generalizability of machine learning, enabling the evaluation of the model’s predictive performance and reliability across diverse datasets from multiple centers, including TCGA, SEER, and ICGC. The comprehensive integration of biological factors, clinical and pathological features, and multi-level indicators in our model substantially enhances its capacity to capture the intricacies of patient survival in HCC.

Our research findings include 11 risk factors, including 7 identified from the TCGA dataset (MYBL2, SF3B4, CDCA8, NUF2, HMMR, P0N1, and PAGE1), and an additional 4 acquired from the SEER database (Age, T, N, M). Several studies have recognized the substantial impact of certain factors on patient outcomes in HCC (44, 45). Our stratified survival analysis revealed that age plays a critical role in determining survival rates, aligning with previous findings. Furthermore, the correlation observed between smaller tumor size and better prognoses in our study underscores the importance of early detection and diagnosis in HCC, as reflected in the Kaplan-Meier curves for T. N and M, which indicate the aggressive progression of HCC, were also found to significantly affect survival rates in our analysis. These findings advocate for a nuanced understanding of HCC, indicating the inadequacy of generic treatment strategies. Moreover, our study validated these risk factors against the National Comprehensive Cancer Network (NCCN) guidelines (11), confirming the model’s emphasis on tumor staging and its reliability.

Apart from established clinical factors, our research innovatively identified 7 genes that influence prognosis. These genes play critical roles in regulating cell-cell interactions, extracellular matrix remodeling, angiogenesis, and inflammatory responses within the tumor microenvironment. For example, MYBL2 plays an important role in regulating the cell cycle, as its high expression is correlated with the staging and grading of various cancers (46). SF3B4 is involved in regulating the cell cycle, cell differentiation, and immune deficiency. Mutations in SF3B4 can lead to abnormal cell growth and contribute to disease development (47). CDCA8 controls the process of cell mitosis and has been identified as an unfavorable prognostic predictor in liver cancer (48). NUF2 participates in chromosome segregation and has been positively correlated with differential immune cell infiltration and various immune biomarkers (49). HMMR is associated with the infiltration levels of neutrophils, CD8+ T cells, and CD4+ T cells in the immune system, as well as the prognosis of patients with cancer (50). PON1 plays a role in cell adhesion and migration, contributing to the regulation of tumor development, oxidative stress, and inflammatory responses (51). PAGE1 is involved in cell apoptosis and immune regulation (52). These gene abnormalities play a role in altering the tumor microenvironment, which impacts the growth, infiltration, and metastasis of HCC. By integrating these differential gene factors into the prognostic risk assessment model, we capture the intricacies of patient survival in HCC.

In addition to assessing the model’s performance in predicting patient survival, our research closely integrates with clinical treatment through the evaluation of patients’ immune infiltration status and their responses to clinical drugs. This enhances our comprehensive understanding of HCC. In recent years, immunotherapy has emerged as a significant breakthrough in HCC treatment, utilizing the patient’s immune system to target tumor cells (53, 54). We examined the association between the model’s predictive results and the immune status by employing GSEA analysis and assessing immune cell infiltration. The results indicated a significant correlation (*P* < 0.05) between high-risk patients and malignant tumor phenotypes, particularly in terms of cell cycle, DNA replication, and immune responses. We have discerned a significant elevation in the infiltration levels of Type2 T helper (Th2) cells within the cohort of high-risk HCC patients (*P* < 0.001), indicating a Th2-dominated immune microenvironment. The cytokines secreted by Th2 cells, such as IL-4 and IL-10, may facilitate tumor growth and assist in the tumor’s evasion of immune surveillance. Therapeutic interventions targeting the Th2 cell pathway, such as PD-1/PD-L1 and CTLA-4 inhibitors, have demonstrated potential in the treatment of other cancers (55, 56). This observation underscores the importance of considering the immune microenvironment when devising therapeutic strategies for HCC.

Additionally, we introduced the novel IPS to assess both the immune system’s activity level and the extent of immune cell infiltration in the tumor microenvironment. This was done with the aim of identifying potential variations in patient response to immunotherapy. The IPS quantifies patients’ potential responsiveness to immunotherapy based on the analysis of expression patterns in immune-related genes. Higher IPS scores generally reflect a more active immune system and an increased likelihood of positive response to immunotherapy (31). We computed IPS scores for patients in both the high-risk and low-risk groups, facilitating a comparison of their immunotherapy responsiveness. The findings showed that the low-risk group exhibited significantly higher responsiveness to immunotherapy (*P* < 0.05), providing theoretical support for the application of immunotherapy in low-risk patients.

Although our research has shown promising results, it is important to acknowledge its limitations. First, the development and prognosis of HCC are influenced by various biological and environmental factors. While we thoroughly considered clinical data and genetic information, it is conceivable that other factors, not accounted for in the model, may also contribute. This underscores the necessity for continual improvement and refinement. Secondly, as our model lacks support from Supplementary Databases, it is advisable to conduct further prospective studies to validate and refine it in relation to immunotherapy and clinical drug responsiveness.

In conclusion, we have successfully developed a machine learning-based prognostic risk model for HCC, providing robust support for personalized treatment strategies in HCC patients. Furthermore, this study highlights the potential importance of utilizing multi-level modeling approaches in the realm of personalized medicine.

## Data availability statement

The data utilized in this study can be obtained by contacting the authors due to restrictions imposed by the data providers, namely the TCGA, SEER, and GSEA databases. Access to these databases is available via their dedicated websites: TCGA (https://portal.gdc.cancer.gov/), SEER (https://seer.cancer.gov/), GSEA (http://software.broadinstitute.org/gsea/index.jsp), and ICGC (https://icgcportal.genomics.cn/). Researchers interested in accessing the data may reach out to the authors for additional information and support in acquiring the required permissions and data access.

## Author contributions

Z-HZ: Data curation, Methodology, Software, Writing – original draft. YD: Supervision, Validation, Writing – review & editing. SW: Investigation, Writing – original draft. WP: Methodology, Supervision, Validation, Writing – original draft, Writing – review & editing.
